# Purification and Molecular Docking Study on the Angiotensin I-Converting Enzyme (ACE)-Inhibitory Peptide Isolated from Hydrolysates of the Deep-Sea Mussel *Gigantidas vrijenhoeki*

**DOI:** 10.3390/md21080458

**Published:** 2023-08-21

**Authors:** Seong-Yeong Heo, Nalae Kang, Eun-A Kim, Junseong Kim, Seung-Hong Lee, Ginnae Ahn, Je Hyeok Oh, A Young Shin, Dongsung Kim, Soo-Jin Heo

**Affiliations:** 1 Jeju Bio Research Center, Korea Institute of Ocean Science and Technology (KIOST), Jeju 63349, Republic of Korea; syheo@kiost.ac.kr (S.-Y.H.); nalae1207@kiost.ac.kr (N.K.); euna0718@kiost.ac.kr (E.-A.K.); junseong@kiost.ac.kr (J.K.); 2 Department of Marine Biotechnology, University of Science and Technology (UST), Daejeon 34113, Republic of Korea; 3 Department of Pharmaceutical Engineering, Soonchunhyang University, Asan 31538, Republic of Korea; shlee80@sch.ac.kr; 4 Department of Food Technology and Nutrition, Chonnam National University, Yeosu 59626, Republic of Korea; gnahn@jnu.ac.kr; 5 Marine Ecosystem and Biological Research Center, Korea Institute of Ocean Science and Technology (KIOST), Busan 49111, Republic of Korea; ohjh@kiost.ac.kr (J.H.O.); shinay@kiost.ac.kr (A.Y.S.); dskim@kiost.ac.kr (D.K.)

**Keywords:** hydrothermal vent mussel, *Gigantidas vrijenhoeki*, angiotensin I-converting enzyme, molecular docking, bioactive peptide

## Abstract

The objective of this study was to prepare an angiotensin I-converting enzyme (ACE)-inhibitory peptide from the hydrothermal vent mussel, *Gigantidas vrijenhoeki*. The *G. vrijenhoeki* protein was hydrolyzed by various hydrolytic enzymes. The peptic hydrolysate exhibited the highest ACE-inhibitory activity and was fractionated into four molecular weight ranges by ultrafiltration. The <1 kDa fraction exhibited the highest ACE inhibitory activity and was found to have 11 peptide sequences. Among the analyzed peptides, KLLWNGKM exhibited stronger ACE inhibitory activity and an IC_50_ value of 0.007 μM. To investigate the ACE-inhibitory activity of the analyzed peptides, a molecular docking study was performed. KLLWNGKM exhibited the highest binding energy (−1317.01 kcal/mol), which was mainly attributed to the formation of hydrogen bonds with the ACE active pockets, zinc-binding motif, and zinc ion. These results indicate that *G. vrijenhoeki*-derived peptides can serve as nutritional and pharmacological candidates for controlling blood pressure.

## 1. Introduction

Hypertension is a major healthcare concern that increases the risk of death, stroke, myocardial infarction, arteriosclerosis, cerebral hemorrhage, and other vascular diseases and is affected by various factors, such as salt intake, smoking, stress, and obesity [1,2]. As part of the complex regulatory system of blood pressure, angiotensin I-converting enzyme (ACE) plays a key role in maintaining blood pressure via the renin-angiotensin-aldosterone system [3]. ACE converts the inactive decapeptide, angiotensin I, by cleaving a dipeptide from the C-terminus to produce an active octapeptide, angiotensin II, a potent vasoconstrictor [4,5]. Moreover, it induces the inactivation of bradykinin, an anti-hypertensive vasodilator, and promotes an increase in blood pressure [6,7]. Therefore, ACE inhibition has become a promising approach for maintaining blood pressure within the normal range.

Several synthetic inhibitors, such as captopril, enalapril, lisinopril, and alacepril, have been developed and used extensively for the management of hypertension and cardiovascular disorders [8,9]. However, the use of these synthetic inhibitors are often accompanied by obvious drug-associated adverse effects, including headaches, insomnia, fever, cough, skin rashes, and increased blood potassium levels [8,10]. Therefore, it has become increasingly necessary to develop therapeutic agents that are free from adverse side effects and to develop effective ACE inhibitors derived from natural sources for the treatment and prevention of hypertension.

Several studies have reported that marine organisms are good sources of protein and contain bioactive peptides with potential biological activities, such as osteoblast differentiation [11,12], anti-cancer [13], antioxidative [14], and anti-inflammatory activities [15]. In particular, bioactive peptides with ACE-inhibitory activity isolated from marine organisms, such as *Takifugu bimaculatus* [16], *Ulva intestinalis* [17], *Paralichthys olivaceus* [18], *Mytilus edulis* [19], and *Perna viridis* [20] have been widely reported. Therefore, research on ACE-inhibitory peptides is of considerable interest to the pharmaceutical industry, and marine organisms are regarded as a promising source of ACE-inhibitory peptides [7]. *Gigantidas vrijenhoeki* is a newly discovered hydrothermal vent mussel species, first reported in 2020, and is known to inhabit the Onnuri Vent Fiedl (OVF) Central Indian Ridge [21,22].

The objective of the present study was to prepare protein hydrolysates and ACE-inhibitory peptides from *G. vrijenhoeki,* and to identify any bioactive peptides with ACE-inhibitory activity. In addition, we investigated the interactions between the bioactive compounds and ACE using molecular simulations.

## 2. Results and Discussion

### 2.1. Approximate Chemical Composition of G. vrijenhoeki

The approximate chemical composition of *G. vrijenhoeki* is presented in Table 1. The major chemical component of *G. vrijenhoeki* was found to be protein, the content of which accounted for 65.83 ± 4.94% of the total dry weight. The lipid, moisture, ash, and carbohydrate contents of *G. vrijenhoeki* were 16.64 ± 0.89%, 2.28 ± 0.04%, 6.29 ± 1.19% and 8.96 ± 0.57%, respectively. Compared to previous studies, the protein content of *G. vrijenhoeki* was found to be higher or similar to *Chlamys farreri* (66.18%) [23], *Mytilus coruscus* (53.2%) [24], and *Perna canaliculus* (43.0%) [25]. Therefore, *G. vrijenhoeki* can be considered to be richer in protein compared to similar species.

### 2.2. Amino Acid Profile of G. vrijenhoeki

The amino acid composition of *G. vrijenhoeki* muscle is listed in Table 2. Glutamic acid (16.39%), aspartic acid (10.20%), glycine (9.01%), and arginine (8.53%) were dominant in *G. vrijenhoeki* muscle. The major amino acids in fish protein and shellfish hydrolysates are glutamic acid, aspartic acid, and glycine [26]. In particular, in *Mytilidae*, such as *M. edulis*, *M. coruscus*, *P. viridis*, and *P. canaliculus*, glutamic acid, aspartic acid, glycine, and arginine are abundant [20,25,27]. In addition, Ijarotimi et al. (2023) reported that glutamic acid serves as a precursor to arginine, which is a precursor for nitric oxide formation that acts as a vasodilator of the arteries, thus lowering blood pressure [28]. Other studies have also reported the ACE inhibitory activity of protein hydrolysates isolated from blue- and green-lipped mussels [19,29,30,31]. Therefore, *G. vrijenhoeki* could be a potential source of ACE inhibitory peptides.

### 2.3. Preparation of GVHs and Their ACE Inhibitory Activity

*G. vrijenhoeki* hydrolysates (GVHs) were obtained by enzymatic hydrolysis with nine proteases, including papain, alcalase, flavourzyme, neutrase, bromelain, protamax, pepsin, trypsin, and α-chymotrypsin, under optimal conditions. The nine hydrolysates were evaluated for their ability to inhibit ACE activity. Among all of the enzymatic hydrolysates, the peptic hydrolysate exhibited the highest level of activity relative to the other enzymatic hydrolysates, with an IC_50_ value of 0.266 mg/mL (Table 3). Compared to previous studies, the ACE inhibitory activity of enzymatic hydrolysates from *G. vrijenhoeki* was more effective than those of seahorse (0.81 mg/mL) [8], Yellowbelly (3.98 mg/mL) [9], scallop (10.28 mg/mL) [32], and blue mussel (1.13 mg/mL) [19] hydrolysates. Interestingly, oysters (0.40 mg/mL) [33] exhibit a similar ACE inhibitory activity.

The peptide hydrolysate from *G. vrijenhoeki* was fractionated by ultrafiltration using membranes of different pore sizes (1 kDa, 5 kDa, and 10 kDa) to obtain fractions of >10 kDa, 5–10 kDa, 1–5 kDa, and <1 kDa. Among all of the fractions, the <1 kDa fraction exhibited the highest ACE inhibitory activity, with an IC_50_ value of 0.025 mg/mL (Table 4). Heo et al. (2017) previously reported that the ACE inhibitory efficiency of a peptide is strongly influenced by the molecular weight thereof. In addition, it has been reported that low-molecular-weight fractions tend to have a more potent ACE inhibitory activity [34,35]. Based on these results, we selected the <1 kDa fraction for further experiments.

### 2.4. Identification of an ACE Inhibitory Peptide

**Figure 1:**
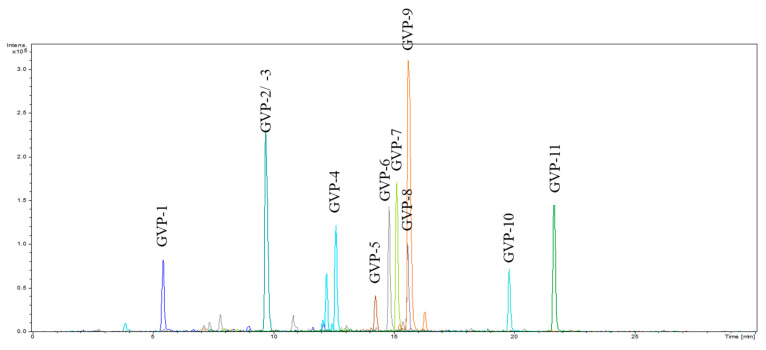
LC-MS chromatogram of <1 kDa fraction from *G. vrijenhoeki* protein.

The molecular masses of the ACE-inhibitory peptides were determined. The <1 kDa fraction was subjected to micro Q-TOF mass spectrometry (MS) and tandem MS analysis, and the results revealed that the fraction was composed of 11 peptides (Figure 1). A synthetic peptide with the same sequence was synthesized and evaluated to validate its ACE inhibitory activity. As shown in Table 5, *G. vrijenhoeki* peptide (GVP)-10 (IC_50_ = 0.007 μM) exhibited stronger ACE inhibitory activity, followed by GVP-7 (IC_50_ = 0.024 μM), GVP-4 (IC_50_ = 0.067 μM), GVP-2 (IC_50_ = 0.162 μM), GVP-3 (IC_50_ = 0.292 μM), GVP-9 (IC_50_ = 0.435 μM), GVP-8 (IC_50_ = 0.513 μM), GVP-5 (IC_50_ = 0.582 μM), GVP-11 (IC_50_ = 0.795 μM), GVP-6 (IC_50_ = 1.390 μM), and GVP-1 (IC_50_ = 2.955 μM). Several reports suggest that the main substrates comprising peptides, such as hydrophobic amino acid residues (aromatic or branched chain) at the C-terminus and positively charged amino acids, are effective for ACE inhibitory activity [1,4,7,30,33,35,36]. This may explain the strong inhibitory activity of GVP-10 (KLLWNGKM) and GVP-7 (ALRPKF), which consist of aromatic amino acids (methionine, M; phenylalanine, F) and positively charged amino acids (lysine, K) at the C-terminus of the analyzed peptide.

### 2.5. Analysis of Molecular Docking Study

**Figure 2:**
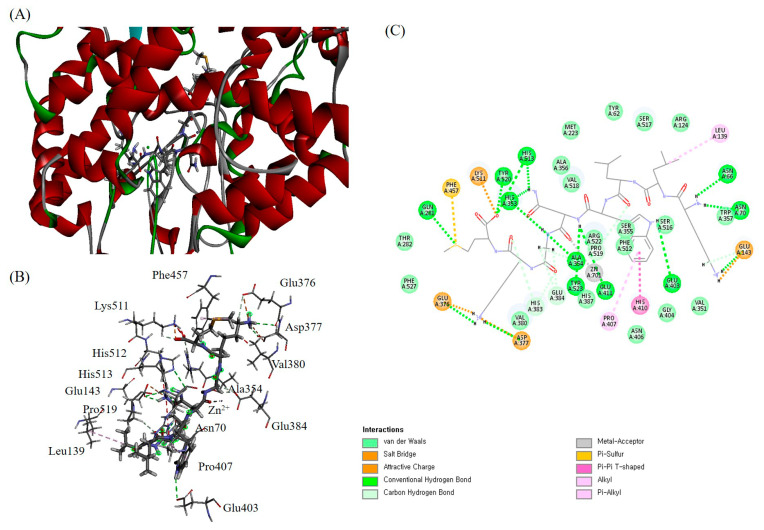
Predicted binding site of GVP-10 with ACE protein (**A**,**B**) and specific points of interaction between GVP-10 and ACE protein (**C**).

Molecular docking studies are effective analytical tools for investigating ligand-protein interactions to understand structure-activity relationships. Therefore, we investigated whether ACE-inhibitory peptides could interact with ACE proteins and inhibit ACE activity, by performing molecular docking analysis using Discovery Studio (Figure 2). ACE consists of three main active site pockets (S1, S′1 and S′2). These pockets are major active sites in ACE and contain different residues. The S1 pocket includes Ala354, Glu384, and Tyr523 residues, the S1′ pocket includes the Glu162 residue, and the S′2 pocket consists of the Gln281, His353, Lys511, His513, and Tyr520 residues [37]. The HEXXH zinc-binding motif is also a main active site, consisting of the His383, Glu384, and His387 residues, and zinc ions [38]. As shown in Table 6, the relative binding energy between GVP-10 and ACE was the highest, indicating that its binding to ACE was the most stable. The binding energy value of GVP-10 was −1317.01 kcal/mol. It was found to interact with the S1 pocket (Ala354, Glu384, and Tyr523), S′2 pocket (Gln281, His353, Lys511, His513, and Tyr520), and zinc-binding motif (His383, His387, Glu411, and zinc ion). Glycine and aspartic acid of GVP-10 were located in the S1 pocket, forming hydrogen bonds with Ala354, Glu384, and Tyr523. Furthermore, methionine of GVP-10 shared hydrogen bond with Gln281, Lys511, His513, and Tyr520 in the S′2 pocket (Figure 2C). Based on these results, GVP-10 could directly interact with the active sites in the S1 and S′2 pockets, thus contributing to its competitive inhibition modalities [39]. In addition, GVP-10 interacted with a zinc-binding motif. Zinc ions play a key role in maintaining ACE activity, and residues in the zinc-binding motif bind to zinc ions to form tetrahedral coordinates [40]. Previous studies have shown that interactions between ACE-inhibitory peptides and the tetrahedral coordination of zinc ions can inhibit ACE activity [41,42]. Therefore, interactions with zinc ions can inhibit ACE inhibitory activity. Among the components of GVP-10, lysine formed a hydrogen bond with His383, and aspartic acid interacted with His411 and zinc ions through a hydrogen bond, facilitating interaction with the zinc-binding motif. Kaewsahnguan et al. (2021) reported that negatively charged amino acids in the ACE active site can interact with zinc ions to lower the catalytic rate through chelation of the critical zinc atom if enzymatic activity occurs [43].

As shown in Figure 2C, the aspartic acid of GVP-10 interacts with the zinc ion and residue Glu411, leading to the distortion of the tetrahedral geometry of ACE. Moreover, the 11 residues surrounding the ACE active site–Ser355, Ala356, Pro407, His410, Phe512, Ser516, Ser517, Val518, Pro519, Arg522, and Phe527 significantly contributed to the stabilization of the ACE inhibitory peptide-ACE complex.

## 3. Materials and Methods

### 3.1. Materials

A deep-sea mussel (*G. vrijenhoeki*) specimen was collected with a video-guided hydraulic grab (Oktopus, Hohenwestedt, Germany) apparatus from the ONNURI vent field in the Indian Ocean (11°14′55.92″ S, 66°15′15.10″ E, at 2014.5 m depth) using the R/V ISABU [21]. The collected sample was immediately rinsed with seawater, directly frozen in a deep freeze, and stored at −80 °C until extraction. Alcalase 2.4 L FG, Neutrase 0.8 L, Flavourzyme 500 MG, and Protamex were purchased from Novo Co. (Novozyme Nordisk, Bagasvaerd, Denmark). Pepsin, trypsin, α-chymotrypsin, bromelain, and papain were purchased from Sigma–Aldrich (St. Louis, MO, USA). All of the other chemicals and reagents used were of analytical grade.

### 3.2. Chemical Composition of G. vrijenhoeki

The chemical composition of *G. vrijenhoeki* was determined as described by Horwitz et al. [44]. Briefly, the crude protein and lipid contents were determined using the Kjeldahl and Soxhlet methods, respectively. The moisture content was determined by placing the sample in a dry oven, and crude ash was prepared at 550 °C in a dry-type furnace.

### 3.3. Amino Acid Composition of G. vrijenhoeki

The amino acid composition was analyzed according to a previously developed high-performance liquid chromatography (HPLC) method [14]. The samples were added to 30 mL of 6 N HCl and incubated for 24 h at 130 °C. The mixtures were filtered with a 0.45 μm syringe filter and used for HPLC analysis. The HPLC system used for the analysis consisted of an Ultimated3000 (Thermo Fisher Scientific, Waltham, MA, USA) and a FL detector 1260FLD (Agilent Technologies, Inc., Santa Clara, CA, USA). Analyses were performed in the binary gradient mode. An Inno C18 column (4.6 × 150 mm, 5 μm, YoungJin Biochrom, Gyeonggi, Korea) was used. The chromatogram was obtained using a fluorescence spectrophotometer at 340/450 nm and 266/305 nm and absorbance at 338 nm.

### 3.4. Preparation of Enzymatic Hydrolysates of G. vrijenhoeki

*G. vrijenhoeki* enzymatic hydrolysis was performed according to a previous method described by Lee et al. [45]. *G. vrijenhoeki* enzymatic hydrolysates were prepared using alcalase, neutrase, flavourzyme, protamex, pepsin, trypsin,-chymotrypsin, bromelain, and papain under optimal conditions (Table 7). Briefly, 1 g *G. vrijenhoeki* and 10 mg of each enzyme were mixed in 100 mL distilled water. The mixtures were then incubated in a shaking incubator for 24 h. After 24 h, the mixtures were incubated at 100 °C for 10 min to inactivate the enzyme, and the pH was adjusted to 7.0. The mixtures were clarified by centrifugation and filtered through Whatman filter paper. The filtered mixtures were lyophilized and kept at −80 °C for further experiments.

### 3.5. Preparation of Molecular Weight Fractionation

GVH was passed through ultrafiltration (UF) membranes (molecular weight cut-offs of 1 kDa, 5 kDa, and 10 kDa) using a laboratory-scale tangential flow filtration (TFF) system (Millipore, Burlington, MA, USA). GVH was subjected to molecular weight fractionation to obtain peptides with molecular weights <1 kDa (1 kDa or smaller), 1–5 kDa (between 1 and 5 kDa), 5–10 kDa (between 5 and 10 kDa), and >10 kDa (10 kDa and larger). All recovered fractions were lyophilized and stored at −80 °C until use.

### 3.6. Identification of ACE Inhibitory Peptide

The molecular masses and amino acid sequences of the purified peptides were determined using a quadrupole time-of-flight mass spectrometer (Micro Q-TOF III mass spectrometer, Bruker Daltonics, Bremen, Germany) coupled with an electrospray ionization (ESI) source. The fraction was separately infused into the electrospray source after being dissolved in distilled water containing 0.1% formic acid, and the molecular mass was determined from the doubly charged [M+2H]^2^ states in the mass spectrum. Following molecular mass determination, peptides were automatically selected for fragmentation, and sequence information was obtained by tandem mass spectrometry (MS) analysis.

### 3.7. Synthesis of the Purified Peptide

The peptide was chemically synthesized at the peptide synthesizer facility of PepTron Inc. (Daejeon, Korea). The peptides were synthesized using the Fmoc solid-phase method with a peptide synthesizer (PeptrEX-R48; Peptron, Inc., Deajeon, Korea). The synthetic peptides were purified using HPCL (Shimadzu, Kyoto, Japan) on a Capcell Pak C18 column (4.6 × 50 mm, 5 μm, Shiseido, Kyoto, Japan). The column was developed at a flow rate of 1.0 mL/min by a linear gradient of acetonitrile containing 0.1% trifluoroacetic acid. The identity of synthetic peptides was confirmed by liquid chromatography-mass spectroscopy (LC-MS) (Shimadzu, Japan), and the purity of the synthetic peptide was confirmed to be over 95%.

### 3.8. ACE Inhibitory Activity Assay

ACE inhibitory activity was measured using the Dojindo ACE Kit-WET kit (Dojindo Laboratories, Kumamoto, Japan), according to the manufacturer’s instructions. The ACE-inhibitory activity was calculated as follows:

$$ACE\;inhibitory\;activity\;(\%)=\frac{A_{control}-A_{sample}}{A_{control}-A_{blank}}\times 100\%$$

where *A_control_* is the absorbance of the positive control, *A_blank_* is the absorbance of the blank containing distilled water, and *A_sample_* is the absorbance of the sample. The IC_50_ value was determined as the concentration of inhibitor required to inhibit 50% of the ACE activity.

### 3.9. Molecular Docking Analysis

The molecular docking analysis was performed according to the method described by Kang et al. [46] with slight modifications. For molecular docking studies, the crystal structure of ACE (PDB code:1O86) was obtained from the protein data bank (PDB; https://www.rcsb.org/, accessed on 11 October 2022). The structures of the 11 peptides derived from GVH were drawn using the CDOCKER tool. Docking of bioactive peptides to ACE was performed using the Lib Dock tool in Discovery Studio 2022 (Biovia, San Diego, CA, USA).

### 3.10. Statistical Analysis

All quantitative data are presented as means ± standard deviation and represent at least three individual experiments conducted using fresh reagents. Statistical comparisons of the mean values were performed using analysis of variance (ANOVA) followed by Duncan’s multiple range test using SPSS software v29. Differences in mean values were considered statistically significant at * *p* < 0.05, ** *p* < 0.01.

## 4. Conclusions

In this study, *G. vrijenhoeki* protein was hydrolyzed using alcalase, neutrase, flavourzyme, protamex, pepsin, trypsin, α-chymotrypsin, bromelain, and papain, and their evaluated ACE inhibitory activity. Among enzymatic hydrolysates, peptic hydrolysate showed the highest ACE inhibitory activity compared to other hydrolysates. Subsequently, the peptic hydrolysate was fractionated by ultrafiltration and their fractions significantly improved the ACE inhibitory activity compared to hydrolysate. The low molecular weight fraction (>1 kDa) showed the highest ACE inhibitory activity and identified eleven ACE inhibitory peptides. Among the identified peptides, GVP-10 (KLLWNGKM) exhibited the strongest ACE inhibitory activity with an IC_50_ value of 0.007 μM. Molecular docking studies indicated that GVP-10 was able to bind to residues in the ACE-active pockets (S1 and S’2), interact with zinc-binding motifs, and coordinate with zinc ions. Based on these results, we propose that the ACE-inhibitory peptide isolated from the *G. vrijenhoeki* protein has a beneficial effect in regulating blood pressure.

## Figures and Tables

**Table 1:** Approximate chemical composition of *G. vrijenhoeki*.

Scientific Name	Protein	Lipid	Moisture	Ash	Carbohydrate
*G. vrijenhoeki*	65.83 ± 4.94	16.64 ± 0.89	2.28 ± 0.04	6.29 ± 1.19	8.96 ± 0.57

**Table 2:** Total amino acids composition of *G. vrijenhoeki*.

Amino Acid	Content (%)
Aspartic acid	10.20
Glutamic acid	16.39
Serine	5.17
Histidine	2.14
Glycine	9.01
Threonine	5.64
Arginine	8.53
Alanine	4.92
Taurine	0.88
Tyrosine	4.04
Valine	4.61
Methionine	2.73
Phenylalanine	3.65
Isoleucine	4.60
Leucine	6.79
Lysine	6.35
Proline	4.35
Total	100.000

**Table 3:** ACE inhibitory activity of enzymatic hydrolysates from *G. vrijenhoeki*.

Enzyme	IC_50_ Value (mg/mL)
Papain	0.401 ± 0.001 ^f^
Alcalase	0.319 ± 0.003 ^d^
Flavourzyme	0.780 ± 0.070 ^i^
Neutrase	0.417 ± 0.010 ^h^
Bromelain	0.402 ± 0.012 ^g^
Protamax	0.281 ± 0.011 ^b^
Pepsin	0.266 ± 0.004 ^a^
Trypsin	0.334 ± 0.001 ^e^
α-chymotrypsin	0.302 ± 0.001 ^c^

The concentration of an inhibitor required to inhibit 50% of ACE activity. The values of IC_50_ were determined by triplicate individual experiments. Means with different letters are significantly different (*p* < 0.05).

**Table 4:** ACE inhibitory activity of molecular weight fractions of peptic hydrolysate from *G. vrijenhoeki*.

Molecular Weight Fraction	IC_50_ Value (mg/mL)
Pepsin hydrolysates	0.266 ± 0.004 ^e^
<1 kDa	0.025 ± 0.022 ^a^
1–5 kDa	0.060 ± 0.006 ^b^
5–10 kDa	0.067 ± 0.001 ^c^
>10 kDa	0.351 ± 0.039 ^d^

The concentration of an inhibitor required to inhibit 50% of ACE activity. The values of IC_50_ were determined by triplicate individual experiments. Means with different letters are significantly different (*p* < 0.05).

**Table 5:** ACE inhibitory activity of peptides from *G. vrijenhoeki* peptic hydrolysate.

Peptide	Peptide Sequence	Molecular Weight (Da)	IC_50_ Value (μM)
GVP-1	KLQE	517.29	2.955 ± 0.165 ^k^
GVP-2	KVLH	496.32	0.162 ± 0.002 ^d^
GVP-3	KVHL	496.32	0.292 ± 0.013 ^e^
GVP-4	LVR	387.27	0.067 ± 0.005 ^c^
GVP-5	PSLVG	472.27	0.582 ± 0.008 ^h^
GVP-6	LNSL	446.26	1.390 ± 0.011 ^j^
GVP-7	ALRPKF	366.23	0.024 ± 0.017 ^b^
GVP-8	PGLADMR	380.19	0.513 ± 0.002 ^g^
GVP-9	LLR	401.28	0.435 ± 0.007 ^f^
GVP-10	KLLWNGKM	495.28	0.007 ± 0.002 ^a^
GVP-11	YALPHAL	392.72	0.795 ± 0.015 ^i^

The concentration of an inhibitor required to inhibit 50% of ACE activity. The values of IC_50_ were determined by triplicate individual experiments. Means with different letters are significantly different (*p* < 0.05).

**Table 6:** Interaction between ACE inhibitory peptide and ACE from molecular docking simulation.

Peptide	Peptide Sequence	Binding Energy (kcal/mol)	ACE Residues
GVP-1	KLQE	−449.06	Glu162, Gln281, His353, Ala354, His383, Lys511, Phe512, His513, Tyr520
GVP-2	KVLH	−992.454	Glu162, Gln281, His353, Ala354, Ser355, His383, Glu384, His387, Glu411, Asp415, Asp453, Lys511, His513, Tyr523, Phe527, Zn^2+^
GVP-3	KVHL	−884.496	Glu162, His353, Ala354, Ser355, His383, Glu384, His387, Asp415, Asp453, Lys511, Phe512, His513, Val518, Arg522, Tyr523, Phe523, Phe527, Zn^2+^
GVP-4	LVR	−570.048	Glu162, Gln281, His353, Ala354, His383, Glu384, His387, Glu411, Asp415, Asp453, Lys454, Lys511, Tyr520, Tyr523, Phe527, Zn^2+^
GVP-5	PSLVG	−684.558	His353, Ala354, Ser355, Ala356, His383, Glu384, His387, Phe391, His410, Glu411, Phe512, His513, Val518, Arg522, Tyr523, Phe527, Zn^2+^
GVP-6	LNSL	−607.594	Glu162, His353, Ala354, Ser355, Ala356, His383, Glu384, His387, Phe391, Glu411, Lys511, Phe512, His513, Val518, Arg522, Tyr523, Zn^2+^
GVP-7	ALRPKF	−670.681	Glu162, His353, Ala354, Ser355, Ala356, His383, Glu384, His387, Phe391, His410, Glu411, Asp415, Asp453, Lys511, Phe512, His513, Ser516, Val518, Tyr523, Phe527, Zn^2+^
GVP-8	PGLADMR	−565.024	Gln281, His353, Ala354, Ser355, Ala356, His383, Glu384, His387, Phe391, Pro407, His410, Glu411, Asp415, Asp453, Lys454, Lys511, Phe512, His513, Val518, Tyr523, Phe527, Zn^2+^
GVP-9	LLR	−540.849	Glu162, His353, Ala354, His383, Glu384, His357, Glu411, Asp415, Asp453, Lys454, Tyr523, Phe527, Zn^2+^
GVP-10	KLLWNGKM	−1317.01	Gln281, His353, Ala354, Ser355, Ala356, His383, Glu384, His387, Pro407, His410, Glu411, Lys511, Phe512, His513, Ser516, Ser517, Val518, Pro519, Tyr520, Arg522, Tyr523, Phe527, Zn^2+^
GVP-11	YALPHAL	−782.256	Gln281, His353, Ala354, Ser355, Ala356, His383, Glu384, His387, Phe391, Pro407, His410, Glu411, Asp415, Asp453, Lys454, Lys511, Phe512, His513, Val518, Tyr523, Phe527, Zn^2+^

**Table 7:** Optimal conditions of enzymatic hydrolysis for various enzymes.

Enzyme	Optimal Conditions	
	pH	Temp. (°C)
Alcalase	8.0	50
Flavourzyme	7.0	50
Neutrase	6.0	50
Protamex	6.0	40
Pepsin	2.0	37
Trypsin	8.0	37
α-chymotrypsin	8.0	37
Bromelain	7.0	50
Papain	7.0	60

## Data Availability

Data is contained within the article.
